# Effect of the COVID‐19 pandemic on diabetic retinopathy and referral levels in the English National Health Service Diabetic Eye Screening Programme

**DOI:** 10.1111/dme.15518

**Published:** 2025-02-03

**Authors:** P. H. Scanlon, C. F. E. Norridge, D. Prentis, N. Holman, P. Rankin, J. Valabhji

**Affiliations:** ^1^ Gloucestershire Retinal Research Group (GRRG) Gloucestershire Hospitals NHS Foundation Trust Cheltenham UK; ^2^ Nuffield Department of Clinical Neuroscience University of Oxford Oxford UK; ^3^ University of Gloucestershire Cheltenham UK; ^4^ NHS England London UK; ^5^ Department of Epidemiology and Bio‐Statistics Imperial College London UK; ^6^ School of Population Health Royal College of Surgeons of Ireland Dublin Ireland; ^7^ Division of Metabolism, Digestion and Reproduction, Imperial College London Chelsea and Westminster Hospital Campus London UK

**Keywords:** COVID‐19, diabetic retinopathy, imaging, retinal screening, screening interval, sight threatening diabetic retinopathy, visual loss

## Abstract

**Aims:**

The aim was to determine the effect of the COVID‐19 pandemic on diabetic retinopathy and referral rates in the English National Health Service (NHS) Diabetic Eye Screening Programme (DESP).

**Methods:**

Non‐patient identifiable data are submitted centrally from the 57 regional centres in the NHS DESP on a quarterly basis and analysed using STATA, comparing 01/04/2019–31/03/2020 and 01/04/2021–31/03/2022. Patient characteristics were analysed from National Diabetes Audit (NDA) data.

**Results:**

There were 2,274,635 grades from the 57 centres in 2019–2020 and 2,199,623 grades in 2021–2022. The proportion of eyes with referable DR increased from 3.1% in 2019–2020 to 3.2% in the 2021–2022 NHS year (*p* < 0.01) with a small increase in the level of non‐referable DR from 24.6% to 24.8% (*p* < 0.01). The median proportion of ungradable eyes in 2019–2020 was 2.6% (IQR: 2.3% to 3.3%) increasing to 3.1% (IQR: 2.5% to 3.7%) in 2021–2022. NDA data demonstrated that the proportions with type 1 diabetes receiving eye screening were higher in the latter year (8.3% vs. 7.3%).

**Conclusion:**

The COVID‐19 pandemic was associated with small increases in referable retinopathy rates from 3.1% to 3.2%, non‐referable DR from 24·6% to 24.8% and an increase in the ungradable image rate from 2.6% to 3.1%, the latter increase possibly being caused by untreated cataract during the pandemic. Risk stratification of invitations in the recovery period was believed to have contributed to keeping the referable rates low and supports a similar approach in extension of the screening interval for low‐risk groups.

**What's new?**

- What is known?
  During the Covid‐19 pandemic progression of diabetic retinopathy was demonstrated due to delayed hospital follow‐up appointments and in a small previous screening study.
- What this study has found?
  The data demonstrate that the COVID‐19 pandemic was associated with small increases in referable retinopathy rates from 3.1% to 3.2%, non‐referable DR from 24·6% to 24.8% and in the ungradable image rate from 2.6% to 3.1%, may have been caused by an increase in numbers with untreated cataract.
- Implications of the study
  This study provides support for extension of the screening interval to two yearly in low‐risk groups.

## 1 INTRODUCTION

The English National Health Service (NHS) Diabetic Eye Screening Programme (DESP) commenced in 2003.^1^ It was reported^2^ in the year 2009–2010 that, for the first time in almost 5 decades, diabetic retinopathy was no longer the leading cause of blindness in working age adults in England and Wales, and a major contributor was considered to be the introduction of the NHS DESP, with further reductions in the following 10 years.^3^

The established pathways^4^ in the English NHS are routine digital screening (RDS), digital surveillance (DS), slit lamp bio‐microscopy (SLB), one of which is offered annually to all people with diabetes in England over the age of 12 years except for a small number who are suspended or excluded.^5^ An invitation to screening could be a fixed appointment or it could be an invitation to make an appointment. All images are graded according to standardised criteria with each eye being classified into an R (retinopathy) and an M (maculopathy) level (Table S1) which determine whether the patient is referred or not. Referable grades are R1M1, R2M0, R2M1, R3aM0, R3aM1 or R3sM1. Non‐referable grades are R0M0, R1M0 and R3sM0.

In 2016, the United Kingdom National Screening Committee (UK NSC) made the recommendation^6^ that a 2‐year screening interval could be implemented within diabetic eye screening (DES). Due to the COVID‐19 pandemic, this change was delayed until 2023, to allow all local DES services to restore their service to pre‐pandemic levels of capacity. However, services did undertake some risk‐stratification in the recovery period according to the guidance issued by Public Health England on 20th May 2020 entitled ‘Risk stratified hierarchy for local services on rescheduling diabetic eye screening during the COVID‐19 response’. This prioritised pregnant women, those who had never been screened and those with retinopathy at their last screening attendance.

The analysis in this study was undertaken to understand the effect on referrals over the COVID‐19 period and assess retinopathy levels in the year 2019–2020 before COVID‐19 and in the 2021–2022 NHS year when most screening programmes had been fully restored.

## 2 METHODS

### 2.1 Study design and data sources

This study used nationwide data from all 57 diabetic eye screening programmes (DESPs) in England who must submit data quarterly according to a pre‐specified data set according to national guidance.^7^ The data were extracted directly using a Visual Basics for Application code from two approved DES management software suppliers in England, NEC OptoMize^8^ and InHealth Intelligence Spectra^9^ to calculate programme performance levels against national standards.^10^

Any programme‐identifiable data were removed before any analysis for this study. The data set contained aggregate data for all grading outcomes for the years 1 April 2019 to 31 March 2020 and 1 April 2021 to 31 March 2022, respectively, across all centres. NHS year quarters are defined as April–June, July–September, October–December and January–March for quarters 1 to 4 (Q1–Q4), respectively.

The 2019–2020 NHS year and its respective grading outcomes were the final year not impacted by the COVID‐19 pandemic and the 2021–2022 NHS year was the first year post‐COVID‐19 onset that risk‐stratified service changes implemented as part of the COVID‐19 response did not apply for the entire cohort of patients. Routine screening was stopped with the national lockdown on 16 March 2020 when there was an instruction to avoid all non‐essential contact. Data from the 2020–2021 NHS year were not included in the analysis of diabetic retinopathy levels because of risk stratification but were included in the analysis of referrals.

To identify whether the characteristics of those screened were different between the two time periods, data from the National Diabetes Audit^11^ (NDA) for 2019/2020 and 2021/2022 were analysed. This provides data on the people who have received eye screening using data recorded in primary care health records and from lists received by eye screening programmes. The standard annual NDA data collection time period is 1 January in year 1 to 31 March in year 2.

### 2.2 Statistical analysis of grading outcomes

Grading outcomes from the 57 English DESPs were analysed from the programme performance reports.

Numbers and proportions of referrals to the Hospital Eye Service (HES) for diabetic retinopathy, splitting these into urgent referrals (HESU) and routine referrals (HESR) from RDS, SLB and DS clinics.

Grading outcomes were assessed and compared between 2019–2020 and 2021–2022 NHS years and defined by the English NHS DESP grading criteria^12^, ^13^ which are shown in Table S1. Each eye is graded with a retinopathy level (R), and the presence or absence of maculopathy (M level) and photocoagulation scars (P level). Any diabetic retinopathy ‘DR’ is defined as the detected presence of any feature(s) of DR. ‘Referable DR’ at screening is defined as the presence of any of the retinal features which constitute NDESP levels R2, R3 or M1. Visual acuity is measured with Snellen or Log MAR which is a logarithm of the minimum angle of resolution. Those people with poor‐quality images are referred for examination by slit lamp biomicroscopy, with numbers and proportions assessed.

To take account of multiple testing, a Bonferroni correction was made on all hypothesis tests. A two‐sample test of proportions was performed to assess changes in the proportions of grading outcomes. An equality of medians test was used to compare the median number of total image sets graded. Cohen's h effect size coefficients were presented with p‐values, where appropriate. Adjusted statistical significance was defined as *p* < 0.01, accounting for a Bonferroni correction to account for multiple testing.

A parameter that we termed the percentage backlog calculation was derived to indicate recovery from delays caused by the pandemic. In this parameter, the denominator was the estimated backlog of screens in each quarter as defined as the mean number of screens pre‐Covid with the addition of any non‐screened eyes from the previous quarter. The numerator was the total number of screens in the quarter. Specific individuals were not able to be followed throughout the time period, so the backlog figures remain as an estimate.

All grading outcomes analysis was performed in STATA 18 (StataCorp. 2023. Stata Statistical Software: Release 18. College Station, TX: StataCorp LLC).

### 2.3 Statistical analysis of the characteristics of those taking up retinal screening

The difference in the median age and duration of diagnosed diabetes between 2019/2020 and 2021/2022 was tested using Mann–Whitney U tests. The difference in the proportions of people attending eye screening by type of diabetes, sex, ethnicity and quintile of socio‐economic deprivation was assessed using chi‐squared tests. Analysis of retinal screening take‐up characteristics was performed in the SAS Enterprise Guide.

### 2.4 Information governance

Data, including NDA and NHS DESP data, are collected and used in line with NHS England's purposes as required under the statutory duties outlined in the NHS Act 2006 and Health and Social Care Act 2012. There is controlled access by appropriately approved individuals to data held on secure data environments entirely within the NHS England infrastructure. Data were processed for specific purposes only, including operational functions, service evaluations and service improvement. The data have been disseminated to NHS England under Directions issued under Section 254 of the Health and Social Care Act 2012. Ethics committee approval is not required for these specific purposes. Exclusion from the NDA is activated by an ‘opt‐out’ system, the National Data Opt‐out Service.^14^ All numbers taken from the NDA are rounded to the nearest 5 to protect individuals' confidentiality. The NHS DESP data are all anonymised and do not contain any linked data.

## 3 RESULTS

Data were available from all 57 diabetic eye screening programmes (DESPs) in England. In 2019–2020, a total of 2,274,635 individuals had their image sets graded and 2,199,623 in 2021–2022.

**FIGURE 1:**
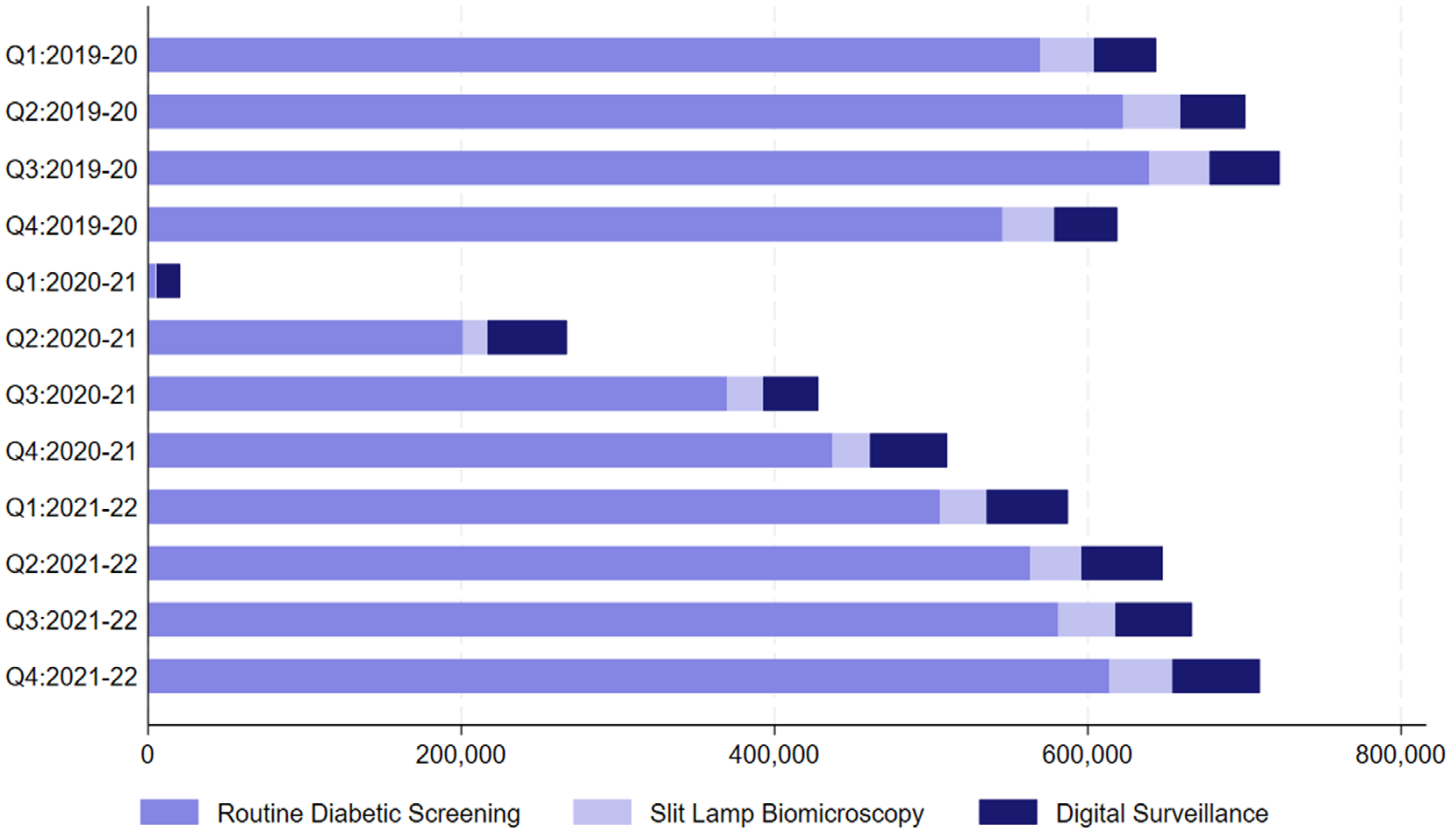
Number of patients attending RDS, SLB and digital surveillance.

The number of appointments in RDS, SLB and DS was lower in the first quarter of the 2020–2021 NHS year compared to previous quarters when routine screening was stopped with the national lockdown on 16 March 2020. There was a percentage decrease of 99.2% and 98.0% for RDS and SLB, respectively, between Q1 of 2019–2020 and Q1 of 2020–2021. The percentage decrease in the number of appointments was less at 62.2% for the DS clinics between the same time periods (Figure 1, Table 1).

**TABLE 1:** The number of referrals from routine diabetic screening, slit lamp bio‐microscopy and digital surveillance clinics.

Number of appointments (column %)^a^	Pre‐COVID (2019–20 NHS year)				COVID (2020–21 NHS year)				Restoration of services (2021–22 NHS year)			
	Quarter 1	Quarter 2	Quarter 3	Quarter 4	Quarter 1	Quarter 2	Quarter 3	Quarter 4	Quarter 1	Quarter 2	Quarter 3	Quarter 4
Total number of people with diabetes	3,423,817	3,459,327	3,520,819	3,538,152	3,540,689	3,553,241	3,572,118	3,582,420	3,617,459	3,648,518	3,682,148	3,732,763
Number excluded	6991	7239	8272	7189	1720	3249	5547	6089	7263	7065	6833	7437
Number suspended (e.g. under HES)	38,773	39,201	46,891	35,812	3528	19,565	34,964	33,833	34,338	32,650	36,537	36,486
Number eligible for RDS screening	2,856,811	2,876,984	2,923,203	2,936,252	2,961,879	2,984,095	2,992,704	3,002,526	3,028,379	3,051,858	3,078,221	3,129,552
Number offered screening	939,606	928,758	1,003,516	846,774	58,288	543,043	832,560	893,048	870,013	931,834	1,032,626	1,066,462
Number RDS (number screened)	569,590	622,606	639,072	531,453	4757	201,133	369,448	437,005	505,407	563,147	581,113	613,887
% of those invited who attended	60.6	67.0	63.7	62.8	8.2	37.0	44.4	48.9	58.1	60.4	56.3	57.6
Number of Referrals (%)												
RDS to DS	10,400 (1.8)	13,386 (2.1)	12,676 (2·0)	10,725 (2.0)	304 (6·4)	8116 (4·0)	15,766 (4·3)	15,059 (3·4)	11,752 (2.3)	11,298 (2.0)	14,286 (2.5)	15,431 (2.5)
RDS to SLB	17,495 (3.1)	17,057 (2.7)	16,129 (2·5)	12,274 (2.3)	174 (3·7)	5480 (2·7)	9946 (2·7)	11,283 (2·6)	13,999 (2.8)	15,641 (2.8)	15,149 (2.6)	15,678 (2.6)
RDS to HESR	10,327 (1.8)	10,773 (1.7)	7738 (1·2)	6170 (1.2)	69 (1·5)	3312 (1·6)	6407 (1·7)	6438 (1·5)	5195 (1.0)	4373 (0.8)	4516 (0.8)	5218 (0.8)
RDS to HESU	1546 (0.3)	1725 (0.3)	1325 (0·2)	1283 (0.2)	66 (1·4)	896 (0·4)	1385 (0·4)	1559 (0·4)	1504 (0.3)	1207 (0.2)	1053 (0.2)	1279 (0.2)
Number SLB	34,358	36,542	38,828	32,858	699	15,713	23,215	24,015	30,056	32,813	36,501	40,158
Number of Referrals (%)												
SLB to DS	167 (0.4)	190 (0.5)	183 (0·5)	110 (0.3)	30 (4·3)	125 (0·8)	134 (0·6)	129 (0·5)	166 (0.6)	202 (0.6)	186 (0.5)	211 (0.5)
SLB to HESR	1424 (3.7)	1114 (3.0)	1242 (3·2)	1104 (3.4)	60 (8·6)	657 (4·2)	1048 (4·5)	1164 (4·8)	1583 (5.3)	1717 (5.2)	1718 (4.7)	1904 (4.7)
SLB to HESU	132 (0.3)	135 (0.4)	94 (0·2)	90 (0.3)	6 (0·9)	70 (0·4)	111 (0·5)	117 (0·5)	121 (0.4)	243 (0.7)	125 (0.3)	114 (0.3)
Number DS	39,553	41,439	44,598	39,174	14,945	50,467	35,295	49,117	51,769	51,890	48,843	55,725
Number of Referrals (%)												
DS to HESR	5162 (13.1)	7604 (18.3)	5654 (12·7)	4864 (12.4)	992 (6·6)	5606 (11·1)	4450 (12·6)	5048 (10·3)	5734 (11.1)	5293 (10.2)	5173 (10.6)	5723 (10.3)
DS to HESU	1145 (2.9)	1652 (4.0)	1296 (2·9)	1156 (3.0)	840 (5·6)	1766 (3·5)	1378 (3·9)	1552 (3·2)	1834 (3.5)	1802 (3.5)	1653 (3.4)	1961 (3.5)

^a^ Column percentages of referrals are calculated within each appointment type. For example, the percentage of referrals from SLB to DS is calculated from the total number of SLB in the time period.

There was a dramatic decrease in the number of patients attending RDS from 531,453 in Q4 of 2019–20 to 4757 in Q1 following cessation of screening on 16 March 2020, increasing to 201,133 in Q2 of 2020–2021. The corresponding numbers attending DS reduced from 39,174 in Q4 of 2019–2020 to 14,945 in Q1, increasing to 50,467 in Q2 of 2020–2021. Many services maintained their DS clinics during Q1 of 2020–2021 even though they stopped their RDS. The number of referrals from RDS and DS into HES routinely (HESR) and urgently (HESU) is shown in Figure S1 that demonstrates the high numbers of referrals from the DS pathway before the main pandemic unfolded and during the pandemic. If one excludes those in the SLB pathway, the routine referrals from digital surveillance were 4864 of a total of 11,034 referrals (44.1%) in Q4 of 2019–2020, 992 of 1061 (93.5%) in Q1 and 5606 of 8918 (62.9%) in Q2 of 2020–2021. The urgent referrals from digital surveillance were 1156 of 2439 (47.4%) in Q4 of 2019, 840 of 906 (92.7%) in Q1 and 1766 of 2662 (66.3%) in Q2 of 2020–2021 (Figure S1).

By the end of the 2021–2022 NHS year, post‐restoration of services, the percentage of eyes with referrals from RDS, SLB and DS in all pathways returned to pre‐COVID‐19 levels.

The number of appointments offered in RDS, SLB and DS, continued to increase throughout the 2021–22 NHS year, reaching the same number of appointments offered in Q4: 2021–2022 as pre‐COVID quarters, Table 1, although we recognise that there would have been an increase in numbers of people with diabetes in that time period.

Despite the number of appointments offered returning to pre‐COVID levels, there were still a small number of services that were still considered to have some backlogs in offering routine (RDS) screening appointments. Eighteen out of 57 services were still considered to have a backlog as of 31 March 2022, with the proportion of appointments unable to be booked within 6 weeks of the expected appointment date varying between 0.4% and 14.0%, with all services recovered by September 2022.

In the description of grades of eyes below this is based on the eye with the most severe level of retinopathy. By the end of 2021–2022, there were only small changes in the percentages of eyes with any DR (R0M0), and the percentage of eyes with referable DR. For eyes receiving a grade R0M0, there were 70.9% of total grades given in 2019–2020 and 70.5% in 2021–2022. There were 26.2% of eyes given a grade with any DR in the 2019–2020 NHS year with 26.4% of eyes with any DR in the 2021–2022 NHS year. The proportion of eyes with referable DR was 3.1% in 2019–2020 and 3.2% in the 2021–2022 NHS year (Table 2).

**TABLE 2:** Changes in the number of patients given each grade in the worst eye from 2019–2020 to 2021–2022 NHS years.

*N* (col %)	2019–20 NHS Year	2021–22 NHS Year
Number of Grades Given	2,274,635	2,199,623
Any DR	594,952 (26·2)	580,721 (26·4)
Referable DR	69,441 (3·1)	69,597 (3·2)
R0M0	1,611,949 (70·9)	1,550,508 (70·5)
R1M0	525,511 (23·1)	511,124 (23·2)
R1M1	49,663 (2·2)	49,936 (2·3)
R2M0	6172 (0·3)	5633 (0·3)
R2M1	7191 (0·3)	7704 (0·4)
R3SM0	1123 (<0·1)	941 (<0·1)
R3SM1	536 (<0·1)	566 (<0·1)
R3AM0	1748 (<0·1)	1705 (<0·1)
R3AM1	3008 (0·1)	3112 (0·1)
Ungradable	67,734 (3·0)	68,394 (3·1)

There was an increase from 69,441 (3.1%) to 69,597 (3.2%) referrals equating to a percentage increase of 0.2% (Table 2). The proportion of referrals varied slightly across centres with a median percentage change of 4.4% (IQR: −6.0% to 16.7%) (Table 2). Of the 57 centres, one centre had an increase of >30% and one centre had a decrease of >30% between the two NHS years (Figure S2).

Overall, there were statistically significant changes in the percentage of grades of referable DR before and after the COVID‐19 pandemic (*p* < 0.01), however, the effect size was lower than Cohen's convention for small effect size (Cohen's h: <0.01). If the rate of referrals had remained the same as the 2019–2020 NHS year, we would expect only 67,151 referrals in the 2021–2022 NHS year. The increase in referrals led to an additional 2437 patients across the country with referable DR.

Of the eyes that were seen to be non‐referable, the proportion of eyes who were graded R1M0 in the 2019–2020 was 24.6% and this showed a small increase to 24.8% in 2021–2022 NHS year (*p* < 0.01; Cohen's *h*: <0.01). There was little variation between centres. The median proportion of eyes across centres was 25.3% (IQR: 23.3%–26.5%) in the 2019–2020 NHS year and 24·9% (IQR: 23.3%–27.3%) in the 2021 NHS year (Table 2).

For the 57 DESPs, the median number of image sets graded within each centre in the 2019–2020 year was 32,592·5 (IQR: 22,073–56,393) compared to the similar median of 30,910 (IQR: 22,428–52,205) in the 2021–2022 NHS year. Overall, the percentage change in the number of image sets graded between 2019–2020 and 2021–2022 was a decrease of 3.3% (*p* < 0.71). The median percentage change in the number of grades between centres was −2.1% (IQR: −11.2%–3.5%). There were three centres with a percentage decrease of >20%, including one centre with a percentage decrease of >30%. There was one centre with a percentage increase of >20% (Figure S3).

Compared to other measures of change in grading outcomes, the differences in the proportion of grades given as ungradable had more variation between centres. The median proportion of eyes graded as ungradable in 2019–2020 was 2·6% (IQR: 2.3–3.3%) and this increased to a median of 3.1% (IQR: 2.5%–3·7%) (*p* = 0.28; Cohen's *h*: <0.01) in the 2021–2022 NHS year. Within centres, the median percentage change in the proportion of eyes deemed ungradable between 2019–2020 and 2021–2022 was 6.8% (IQR: −12.2% to 31.7%). There were six centres that had a decrease in the number of ungradable images of >30% from the 2019–2020 to 2021–2022 NHS years. There were 16 centres who had an increase in ungradable images >30% from the 2019–2020 to 2021–2022 NHS year. There was one centre that had an increase in over 125% of ungradable images between the 2 years, which has been highlighted in red in Figure S4.

A total of 3,644,725 and 3,799,890 people aged 12 years or older were included in the 2019/2020 and 2021/2022 National Diabetes Audits, respectively. The number of people with a record of having received eye screening was 2,739,775 (75.2%) between 1 January 2019 and 31 March 2020 and 2,452,750 (64.5%) between 1 January 2021 and 31 March 2022. The median age of people receiving screening in 2019/20 was 65 years (IQR 53–75) and 64 years (IQR 53–75) in 2021/2022 (*p* < 0.005). Median duration was 7 years (IQR 2–14) in 2019/20 and 7 years (IQR 3–15) in 2021/2022 (p < 0.005). The proportion of people who received eye screening who had type 1 diabetes was higher (8.3% vs. 7.3%) and the proportion with type 2 diabetes was lower (90.8% vs. 92.1%) in 2021/22 than in 2019/20 (*p* < 0.005). There were statistically significant but minimal differences in the proportion of people who received eye screening by quintile of socio‐economic deprivation and by ethnicity (*p* < 0.005 for both characteristics) (see Table 3).

**TABLE 3:** Proportion of people receiving eye screening by type of diabetes, socio‐economic deprivation and ethnicity, 2019/20 and 2021/22 (15‐month time periods).

	2019/2020		2021/2022	
	*n*	%	*n*	%
Type of diabetes				
Type 1 diabetes	200,280	7.3	205,515	8.3
Type 2 diabetes	2,538,480	92.1	2,246,225	90.8
Other or unknown types of diabetes	1015	0.6	1010	0.9
Socio‐economic deprivation				
Most deprived	630,675	22.9	560,215	22.6
Second most deprived	611,260	22.2	548,305	22.2
Third most deprived	570,245	20.7	514,765	20.8
Second least deprived	513,640	18.6	463,040	18.7
Least deprived	420,750	15.3	379,550	15.3
Missing	8740	0.3	8820	0.4
Ethnicity				
White	1,974,125	71.6	1,757,245	71.0
Mixed	28,055	1.0	28,090	1.1
Asian	361,315	13.1	342,840	13.9
Black	123,980	4.5	123,705	5.0
Other	51,835	1.9	48,515	2.0
Unknown	215,995	7.8	174,295	7.0

## 4 DISCUSSION

These analyses have demonstrated small increases in grades of retinopathy attained via the NHS DESP in people with diabetes in England in the post‐COVID‐19‐onset year 2021–2022 when services were being restored compared to the pre‐COVID‐192019–2020 year. Although the numbers are large and the effect size is small, the total number of screenings is also slightly smaller. These results are therefore compatible with the expected small increase in progression of those with background DR, with 70% of the cohort having no diabetic retinopathy at baseline. Comparison of demographic characteristics between 2 year cohorts shows that there were proportionally slightly more people with type 1 diabetes screened, and the median age of those screened was slightly younger, with no meaningful differences in other characteristics.

The disparity in numbers receiving eye screening between the English NHS DESP figures and the NDA figures is mostly because the English NHS DESP provides data over the 12‐month period from 1 April in year 1 to 31 March in year 2 and the NDA provides data over a 15‐month time‐period from 1 January in year 1 to 31 March in year 2.

The number of people with diabetes continues to rise but the number of appointments offered in the 2021–22 year had not risen and the attendance rates were disappointing at 56%–60%. This may have been because there were still some concerns of patients in attending these appointments in the aftermath of the Covid‐19 pandemic. There was considerable variation between centres but, as the data were anonymised, we did not wish to speculate on the reasons. It is uncertain why the proportions with type 1 diabetes receiving eye screening were higher in the latter year (8.3% vs. 7.3%).

The strengths of this study are the large numbers with whole national diabetes population data. The limitations are that data are not linkable at the individual patient level, the use of different data sources (NHS DESP and NDA data) and slightly different time periods (12 vs. 15 months) to compare patient characteristics and the limitation of the uniqueness of the NHS DESP.

The COVID‐19 pandemic resulted in significant mortality directly and indirectly related to the infection.^15^, ^16^ Romero‐Aroca reported^17^ on a screening study that reported a slight increase in cases of the most severe forms of DR, beginning in the year 2021.

Screening services made a conscious effort to continue the DS pathway, which contains the more severe retinopathy levels R1M1, R2M0 and R2M1, during the Covid‐19 pandemic. The DS pathway became an even higher contributor to the referral rate with 992 of 1061 (93.5%) routine referrals and 840 of 906 (92.7%) urgent referrals in Q1 of 2020–2021.

Screening services used the previous research on the risk of progression of diabetic retinopathy in pregnancy,^18^ in those who had never been screened^19^ and those with retinopathy at their last screening attendance^20^ to prioritise invitations in the Covid recovery phase. This stratification was similar to what the NHS Diabetic Eye Screening Programme had recommended for their extension of the screening interval in low‐risk groups. The proportion of eyes with referable DR only increased from 3.1% in 2019–2020 to 3.2% in the 2021–2022 NHS year (*p* < 0.01; Cohen's *h*: <0.01) which endorsed this stratification approach. There was a small increase in the level of nonreferable DR from 24.6% to 24.8% (p < 0.01; Cohen's h: <0.01). This is not surprising, given the reports^16^ of reduction in performance of the eight care processes of monitoring diabetes in primary care in England during the pandemic and the reported association^21^ with incident diabetic retinopathy.

The median proportion of eyes graded as ungradable in 2019–2020 was 2·6% (IQR: 2.3%–3.3%) and this increased in 2021–2022 to a median of 3.1% (IQR: 2.5%–3.7%) with a doubling in some centres. The cessation and reintroduction of cataract surgery/services during and following the pandemic, and regional variations, may have accounted for the increases.

The English NHS DESP recovered from the pandemic quicker than many other services in England with all services recovered to their previous invitation levels by September 2022 with further increases in invitation numbers over the following 12 months in line with the increased number of people with diabetes. There is very strong oversight of screening by the regional programme boards and, services that were not recovering were having to give regular progress reports and timelines for improvement to NHS England. This helped to stimulate the increased activity.

To conclude, there was some evidence that the COVID‐19 pandemic and resulting lifestyle, healthcare and societal changes were associated with increases in referable retinopathy rates from 3.1% to 3.2% and in the level of non‐referable DR from 24.6% to 24.8% in the screening population in England. However, these changes were small and, while statistically significant, in terms of usual year‐on‐year variation, were not necessarily clinically significant. This suggests that the prioritisation of the higher‐risk groups in the screening recovery phase of the pandemic was successful and provides further support for the planned introduction of extended screening intervals for the English NHS DESP, which uses a very similar risk stratification approach.

## FUNDING INFORMATION

The funding for routine data collection within the English NHS DESP was from Public Health England. NIHR‐support funding supported the statistical analysis of the NHS DESP data. NHS England supported the NDA analysis.

## CONFLICT OF INTEREST STATEMENT

J.V. was the National Clinical Director for Diabetes and Obesity at NHS England from April 2013 to September 2023 and is also supported by the North‐West London NIHR Applied Research Collaboration and the Imperial NIHR Biomedical Research Centre. DP is an analyst at NHS England and is involved in the analysis of the NHS Diabetic Eye Screening Programme data. PR is the Programme Manager for the English NHS DESP at NHS England. PS is the Clinical Lead/Advisor for the English NHS DESP and is a Consultant Ophthalmologist at the Gloucestershire Hospitals (GH) NHS FT and at the Oxford University Hospitals NHS FT. PS declares that in the last 12 months, he has received speaker fees from Topcon and Bayer, expenses from Boehringer and Bayer, and his research team have received unrestricted research grants from Optos, Zeiss and Centervue Ltd. CN is a statistician working with PS at GHNHSFT. NH is funded by NHS England to undertake research using the National Diabetes Audit.

## Supporting information


**Table S1.** English NHS DESP Grading Classification.

**Figure S1.** Routine Referrals to the Hospital Eye Service (HESR) and Urgent Referrals (HESU) from RDS and DS between 2019 and 2021.

**Figure S2.** The percentage change in the number of referrals in each centre between 2019–2020 and 2021–2022.

**Figure S3.** The percentage change in the number of image sets graded between 2019–2020 and 2021–2022.

**Figure S4.** The percentage change in the number of image sets graded as ungradable in 2019–2020 and 2021–2022, with the centre who had an increase of >125% highlighted in red.
