# Supplementary material for: Effect of the COVID‐19 pandemic on diabetic retinopathy and referral levels in the English National Health Service Diabetic Eye Screening Programme
Source: Diabet Med. 2025 Feb 3;42(5):e15518. doi: 10.1111/dme.15518 (PMC12006553; doi:10.1111/dme.15518)
Supplement: Supplementary file 1 — Table S1. English NHS DESP Grading Classification. [file DME-42-e15518-s002.docx]

**Supplementary Table 1 - English NHS DESP Grading Classification**

| **R** |  | **Retinopathy** (English NHS DESP Programme Grading Classification) | |
| --- | --- | --- | --- |
| R0 | None | No DR |  |
| R1 | Background | microaneurysm(s) or HMa* |  |
|  |  | retinal haemorrhage(s) |  |
|  |  | venous loop |  |
|  |  | any exudate in the presence of other non-referable features of DR |  |
|  |  | any number of cotton wool spots (CWS) in the presence of other non-referable features of DR |  |
|  |  |  |  |
| R2 | Pre-proliferative | venous beading |  |
|  |  | venous reduplication |  |
|  |  | multiple blot haemorrhages |  |
|  |  | intraretinal microvascular abnormality (IRMA) |  |
|  |  |  |  |
| R3 | Proliferative | R3a (Active Proliferative Retinopathy) | All newly occurring R3 patients with: |
|  |  |  | new vessels on disc (NVD) |
|  |  |  | new vessels elsewhere (NVE) |
|  |  |  | pre-retinal or vitreous haemorrhage |
|  |  |  | pre-retinal fibrosis ± tractional retinal detachment |
|  |  | R3s (Stable post treatment) | Evidence of Peripheral Retinal Laser Treatment  AND |
|  |  |  | Stable retina from photograph taken at or shortly after discharge from the Hospital Eye service (HES) |
| **M** |  | **Maculopathy** |  |
| M0 |  | No maculopathy | absence of any M1 features |
| M1 |  | exudate within 1 disc diameter (DD) of the centre of the fovea | M1 - list of features as now - but individual features are not mutually exclusive |
|  |  | group of exudates within the macula |  |
|  |  | retinal thickening within 1DD of the centre of the fovea (if stereo available) |  |
|  |  | any microaneurysm or haemorrhage within 1DD of the centre of the fovea only if associated with a best VA of ≤ 6/12 (if no stereo) |  |
| **P** |  | **Photocoagulation** |  |
|  |  | No evidence of previous photocoagulation | No grade is assigned |
| P |  | focal/grid to macula or peripheral scatter | Only assigned if laser scars are identified |
| **U** |  | **Unclassifiable** |  |
|  |  | An image set that is inadequate for grading |  |
| *HMa is a term used when it is difficult to tell the difference between a microaneurysm and a dot haemorrhage. | | | |
