# Supplementary figures and images for: Effect of the COVID‐19 pandemic on diabetic retinopathy and referral levels in the English National Health Service Diabetic Eye Screening Programme
Source: Diabet Med. 2025 Feb 3;42(5):e15518. doi: 10.1111/dme.15518 (PMC12006553; doi:10.1111/dme.15518)

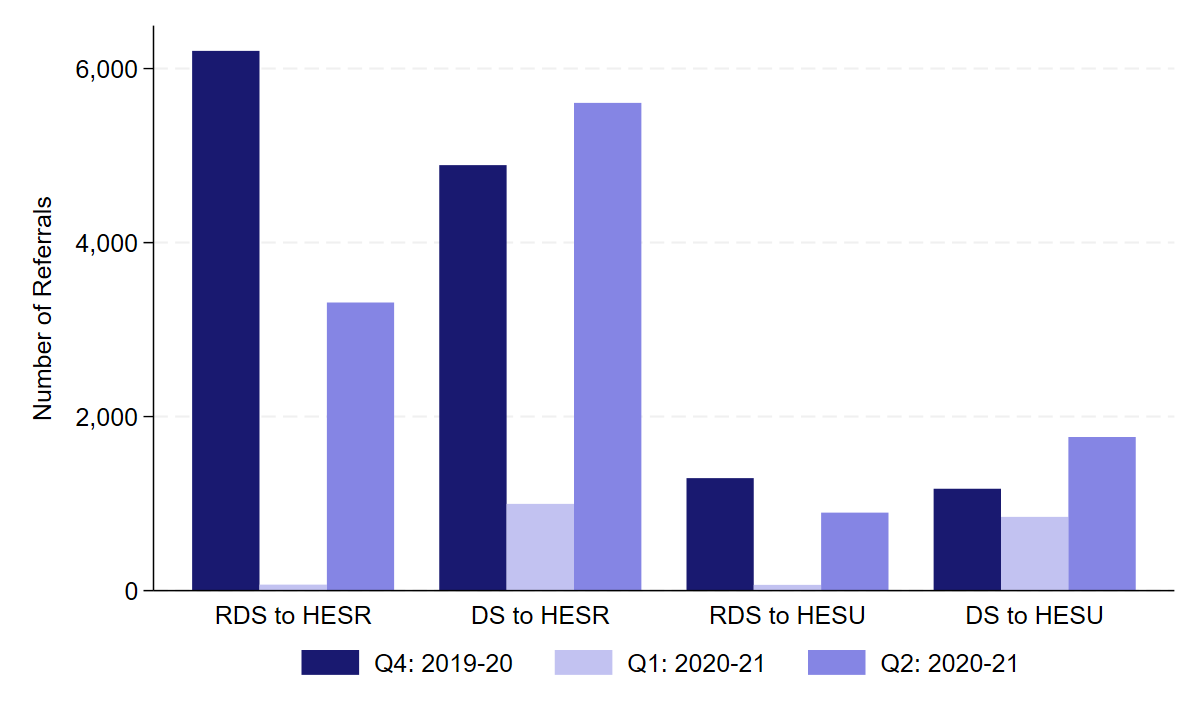

Supplement: Supplementary file 2 — Figure S1. Routine Referrals to the Hospital Eye Service (HESR) and Urgent Referrals (HESU) from RDS and DS between 2019 and 2021. [file DME-42-e15518-s001.tif]

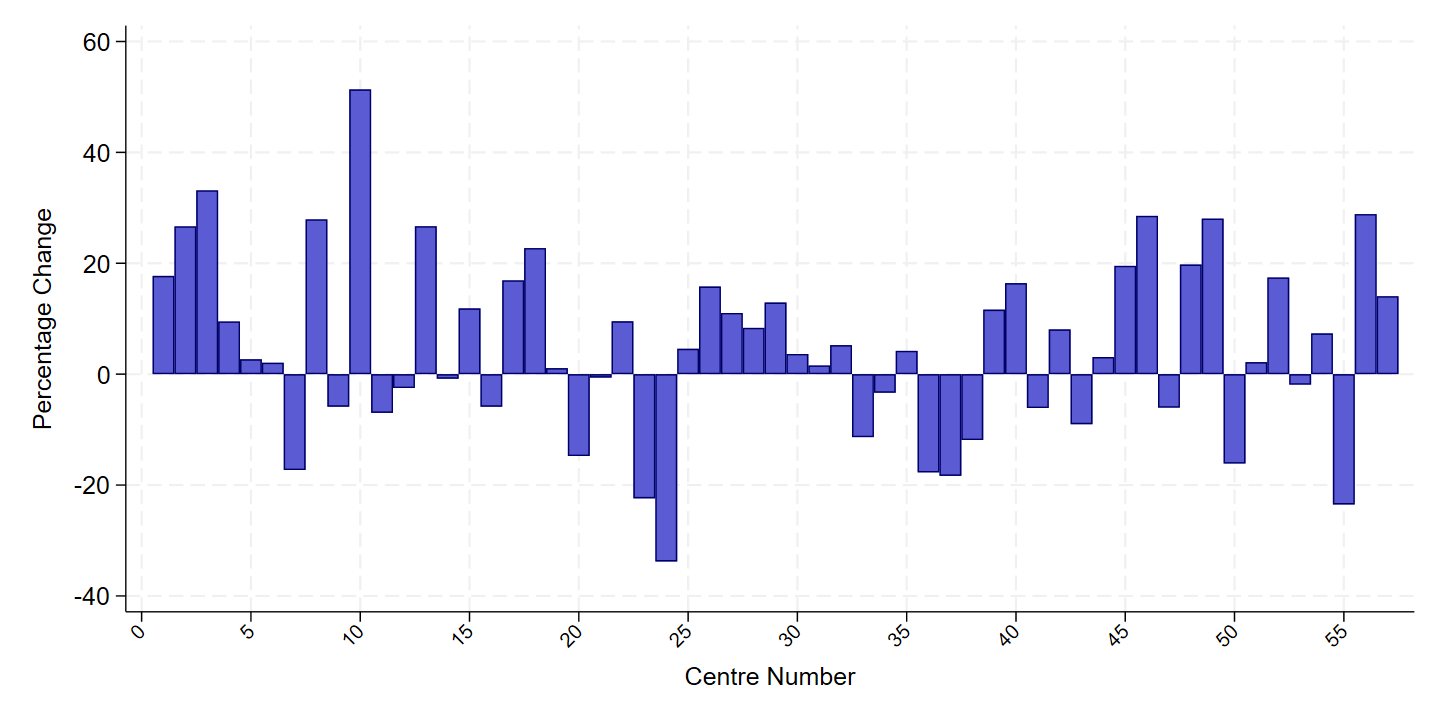

Supplement: Supplementary file 3 — Figure S2. The percentage change in the number of referrals in each centre between 2019–2020 and 2021–2022. [file DME-42-e15518-s005.tif]

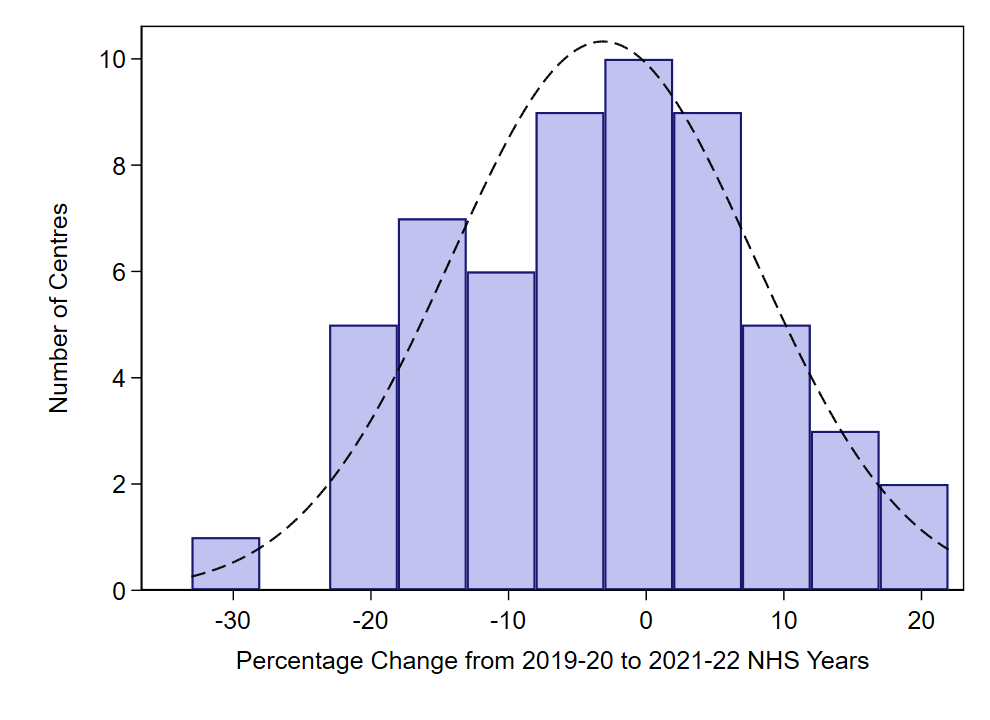

Supplement: Supplementary file 4 — Figure S3. The percentage change in the number of image sets graded between 2019–2020 and 2021–2022. [file DME-42-e15518-s004.tif]

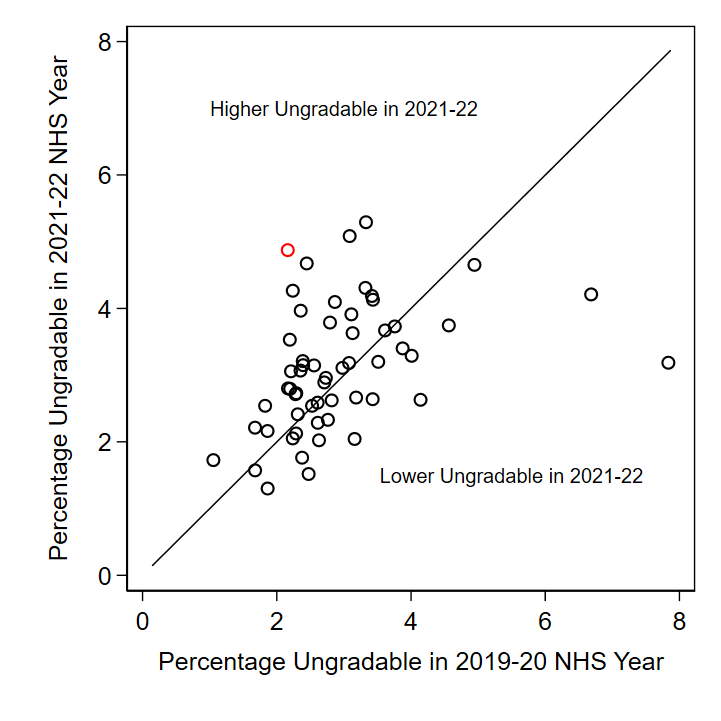

Supplement: Supplementary file 5 — Figure S4. The percentage change in the number of image sets graded as ungradable in 2019–2020 and 2021–2022, with the centre who had an increase of >125% highlighted in red. [file DME-42-e15518-s003.tif]
